# The long way from bench to bedside

**DOI:** 10.1186/1746-160X-8-S1-I17

**Published:** 2012-05-25

**Authors:** Olivier Gaide

**Affiliations:** 1 Department of Dermatology, Geneva University Hospital, Switzerland

## 

Loss-of-function mutations in the ectodysplasin A gene, *EDA*, have been associated with X-linked hypohidrotic ectodermal dysplasia (XL-HED) since 1996. In 2003, we made use of this information to engineer a recombinant soluble form of ectodysplasin A that has the potential to revert the disease in mice. Almost 10 years later, a cure is still not available for individuals affected by the disease. Is this normal? Part of the answer lies in the complexity of the process linking the proof of principle (in mice) and the authority-approved human treatment. It involves the production of a clinical grade pharmacological drug, a tricky process that requires clean-from-the-start-engineering and extensive stability/efficacy/toxicity controls at every step. And this is only the beginning. Defining treatment windows, regimens, doses and biomarkers of efficacy requires significant research efforts; often tedious and unlikely to be published in high-impact journals. Production and research must run in parallel to discussions with regulatory agencies, each process influencing the other. Finally, a wealth of clinical data must be put together, including precise natural history, identification and preparation of clinical centers, clinical trial protocols set up and filing. Taken together, these steps require two things: i) time and ii) dedicated professionals from many different fields: scientists, clinicians, pharmacologists, production experts, regulatory experts and, importantly, a strong industrial partner. It is a fascinating process, subject to delays and pitfalls, but essentially sound as it drives for the development of the safest and best drug possible.

